# Knowledge, attitude and practice on screening and early diagnosis of prostate cancer of primary health care providers in the Free State

**DOI:** 10.4102/phcfm.v15i1.3688

**Published:** 2023-02-28

**Authors:** Matthew O.A. Benedict, Wilhelm J. Steinberg, Frederik M. Claassen, Nathaniel Mofolo, Cornel van Rooyen

**Affiliations:** 1 Department of Family Medicine, Faculty of Health Sciences, University of the Free State, Bloemfontein, South Africa; 2 Department of Urology, Faculty of Health Sciences, University of the Free State, Bloemfontein, South Africa; 3 Faculty of Health Sciences, School of Clinical Medicine, University of the Free State, Bloemfontein, South Africa; 4 Department of Biostatistics, Faculty of Health Sciences, University of the Free State, Bloemfontein, South Africa

**Keywords:** Knowledge, attitude and practice, primary health care practitioners, primary health care providers, prostate cancer screening, early diagnosis

## Abstract

**Background:**

Prostate cancer is topmost in terms of incidence and mortality among men in sub-Saharan Africa, including South Africa. Prostate cancer screening is beneficial only to certain categories of men, making a rational screening approach necessary.

**Aim:**

This study aimed to assess the knowledge, attitudes and practice (KAP) regarding prostate cancer screening among primary health care (PHC) providers in the Free State, South Africa.

**Setting:**

Selected district hospitals, local clinics and general practice rooms.

**Methods:**

This was a cross-sectional analytical survey. Participating nurses and community health workers (CHWs) were selected through stratified random sampling. All available medical doctors and clinical associates were approached to participate, totalling 548 participants. Relevant information was obtained from these PHC providers using self-administered questionnaires. Both descriptive and analytical statistics were computed using Statistical Analysis System (SAS) Version 9. A *p*-value < 0.05 was considered significant.

**Results:**

Most participants had poor knowledge (64.8%), neutral attitudes (58.6%) and poor practice (40.0%). Female PHC providers, lower cadre nurses and CHWs had lower mean knowledge scores. Not participating in prostate cancer–related continuing medical education was associated with poor knowledge (*p* < 0.001), negative attitudes (*p* = 0.047) and poor practice (*p* < 0.001).

**Conclusion:**

This study established appreciable KAP gaps relating to prostate cancer screening among PHC providers. Identified gaps should be addressed through the preferred teaching and learning strategies suggested by the participants.

**Contribution:**

This study establishes the need to address KAP gaps regarding prostate cancer screening among PHC providers; therefore necessitating the capacity-building roles of district family physicians.

## Introduction

Noncommunicable diseases (cancers inclusive) are responsible for about 70% of all deaths worldwide, with the majority of these deaths occurring in the low- and middle-income countries.^1^ Hence, the Sustainable Developmental Goal 3.4 aims ‘to reduce, by one third, premature mortality from non-communicable diseases through prevention and treatment and promote mental health and well-being by 2030’.^2^

In sub-Saharan Africa, prostate cancer (PCa) is topmost in terms of incidence and mortality among men.^3^ Both foreign^4,5^ and local^6,7^ studies have shown racial disparities in PCa presentation, with men of African ancestry being mostly affected. In South Africa, black men often present late and with advanced disease, thus contributing to an increased mortality and morbidity. Poverty, poor socio-economic status, illiteracy and a lack of knowledge of the disease symptoms are some of the factors responsible for late presentation.^8^ Most South African indigenous languages do not even have translations for the term ‘prostate’.^8^

The Free State has the lowest life expectancy (male 55 years; female 61 years) compared with the average figures for the other provinces in South Africa (male 62 years; female 68 years).^9^ The black race is mostly associated with poor socio-economic status and concomitant poor health outcomes. There is therefore the need to prioritise access to basic primary health care (PHC) services, such as screening, in the study setting.

Relevant international^10^ and local^11^ associations have recognised some benefits of PCa screening among certain categories of men and have therefore published guidelines for screening. Screening for PCa has been controversial because of over-diagnosis, overtreatment and certain accompanying side effects such as sexual and urinary dysfunction.^12^ However, a recent study on the harm-to-benefit of PCa screening showed that the potential for over-diagnosis and overtreatment notwithstanding, the net benefit of prostate-specific antigen (PSA) screening is greater for black men than the general population. This may indicate the need for race-specific screening guidelines.^13^ The United States Preventive Task Force (USPSTF) has reported a potential benefit of decreasing deaths from PCa in men aged 55–69 years with PSA screening; this is doubtful for men above 70 years of age.^14^

South African guidelines^11^ recommend that PCa screening (PSA and digital rectal examination [DRE]) be performed in all men from 45 years onwards in the absence of identifiable risk factors and from 40 years in black men, where there is a family history of PCa and other identifiable risk factors. Yet there is evidence that African men are less likely to participate in PCa screening with a view to making an early diagnosis, which may in part explain the disparity in PCa deaths.^15^ Poor adherence to screening guidelines among black men could indicate gaps in their knowledge, culture and beliefs regarding PCa and related aspects.^15^ In a review study, barriers related to PCa screening among men in sub-Saharan Africa were related to client, healthcare provider and healthcare systems factors.^16^ The influence of healthcare providers is an important factor associated with PCa screening uptake among men.^17,18^

According to the health belief model, individual beliefs and certain sociopolitical factors are strong determinants of health behaviours.^19^ Healthcare providers support users to make healthcare choices through shared decision-making (SDM).^20^ Guidelines of the American Cancer Society highlighted the following as vital information needed for men to make screening decisions: risks, potential benefits and uncertainties regarding PCa screening.^21^ Ng and Lee^22^ highlighted six steps in the practice of SDM: (1) identify the decision, (2) list the available options, (3) discuss the pros and cons of each option, (4) elicit patient values, (5) provide support and (6) make a decision. More so, it has been shown that healthcare users from socio-economically disadvantaged areas prefer SDM.^20^

In a European study that compared general practitioners (GPs) and urologists in their handling of PSA testing and guidelines on early detection of PCa, unlike the urologists, the GPs perceived the PSA test not to be useful. In addition, the GPs showed a less proactive approach to informing men about PSA and were less familiar with guidelines and recommendations on PSA testing.^23^ This might imply a knowledge gap among these GPs. A United States (U.S.) study showed a poor practice of SDM among some physicians involved with PCa screening with PSA.^24^ At the 8th International Prostate Cancer Congress, better education of primary care physicians on SDM was recommended to achieve an improved approach to PCa screening.^25^ In a Malaysian study, more than half of the GPs overestimated the positive predictive values of PCa screening tools. The same study also showed that GPs who would consider having a PSA test performed on themselves were more likely to screen asymptomatic men than GPs who would not have the test.^26^

In a study that assessed the knowledge and attitude of primary care physicians in Florida, U.S., regarding PCa screening, the mean knowledge score was 66%. More than 70% of the responders believed that screening was effective, and the attitude scores were associated with practice setting and the proportion of patients having medical aid.^27^ Primary health care providers’ knowledge and attitudes towards PCa screening may affect their approach to the screening of men. Studies exploring this subject are, however, scarce in our study setting. Foreign studies^28,29^ have shown the roles of nurses and community healthcare workers in health promotion and SDM relating to PCa screening. These roles should be encouraged among these nonmedical PHC cadres, who are first-line PHC providers in South African local communities and clinics.

As explained here, the controversy associated with PCa screening may inform the varied attitude and practice of PHC providers. Studies have identified gaps in knowledge, attitude and practice (KAP) regarding PCa screening among men; however, there is a scarcity of local studies on this subject among PHC providers in South Africa.

Therefore, the aim of the study was to assess the KAP regarding PCa screening and early diagnosis among PHC providers in the Free State, South Africa. The objectives were to determine: (1) demographic details, (2) KAP on PCa screening and early diagnosis, (3) participants’ perceived need for enhanced knowledge on PCa screening and their preferred learning methods and (4) factors associated with participants’ KAP.

## Research methods and design

### Study design

This was a cross-sectional analytical survey carried out among PHC providers in the Free State, South Africa, over six months (01 January 2021 – 30 June 2021).

### Study setting and rationale for this setting

The study setting chosen for this study was the Free State, which is geographically the third-largest province in South Africa, constituting 5.1% of the national population. The estimated population was 2 971 708 as of 2019. This province comprises Mangaung Metropolitan Municipality (MMM) and four district municipalities: Xhariep, Lejweleputswa, Thabo Mofutsanyana and Fezile Dabi. These district municipalities are subdivided into 19 local municipalities. The economy is dominated by agriculture, mining and manufacturing.^30^

The PHC facilities in the Free State include 231 PHC clinics, that is, local clinics and community health centres, and 24 district hospitals. Most of the population using public health services attend these healthcare facilities. Ward-Based Primary Health Care Outreach Teams (WBPHCOTs) are linked to the PHC facilities and consist of community health workers (CHWs) led by nurses. The WBPHCOTs undertake home visits, assess the health status of individuals in households and provide health education and promotion services; they identify and refer those in need of preventive, curative or rehabilitative services to relevant PHC facilities.

Mangaung Metropolitan Municipality of the Free State is one of the top populated black townships in South Africa.^31^ According to an unpublished retrospective cross-sectional study conducted in Bloemfontein, Free State, African men were shown to be at higher risk of having PCa of worse prognosis, that is, Gleason score ≥ 8, higher mean PSA levels and more advanced local staging, compared with their European counterparts.^32^

### Target population

The target population comprised PHC workers (doctors, nurses, clinical associates and CHWs) in the Free State. The PHC workers include all cadres of state-employed medical doctors (interns in Family Medicine rotation, community service medical officers, medical officers, Family Medicine registrars and specialist family physicians) and GPs in private practice, nurses (professional, enrolled and enrolled nursing assistants), clinical associates and CHWs working in the PHC clinics. The majority of these PHC providers (especially the nurses and CHWs) work in the community and are not hospital-based. The subject of PCa screening, like any other screening, falls within PHC services and does not require specialised capabilities. Healthcare providers working in more specialised settings were excluded from this study.

### Sample and sampling

Owing to the diversified cadres of the study population, multiple sampling methods were engaged to maximise the sample size per cadre of PHC provider while also minimising the risk of bias. The total number of PHC medical doctors in the Free State at the time of the study was less than 200; hence, they were all targeted without sampling. The same went for clinical associates, who are less than 20 in the province. On the other hand, the PHC nursing staffs and CHWs in the Free State were much more numerous; hence, a stratified sampling was engaged for these cadres.

All state-employed medical doctors and clinical associates were easily accessed through the corresponding PHC facilities in the Free State (i.e. district hospitals and PHC clinics). Therefore, they were all approached to participate. There were approximately four nurses per 231 fixed PHC clinics (total of about 924 nurses) and around two full-time CHWs per 231 fixed PHC clinics (a total of about 462 CHWs). With the aid of the Raosoft sample size calculator (Raosoft, Inc., Seattle, Washington, United States),^33^ setting the margin of error at 5%, confidence level at 95%, response distribution at 50% and with the population sizes of 924 and 462, the authors arrived at sample sizes of 272 and 210, respectively, for nurses and CHWs.

The list of the clinics was obtained from each district office. Through the stratified simple random sampling method, 105 clinics in total were selected from the five districts in the Free State, that is, 21 clinics per district. Three nurses and two CHWs were targeted from each of the 105 selected clinics.

### Measurement, data collection and questionnaire

For the purpose of this study, data were obtained from primary sources, that is, the eligible PHC providers in the Free State. A self-administered questionnaire was used. The questionnaire was adapted from previous similar surveys^26,34,35^ aimed to understand the KAP of primary care physicians towards PCa and PCa screening. The researcher and his (research) assistant visited the selected PHC facilities and administered the questionnaires to consenting participants. An electronic version of the questionnaire was e-mailed to GPs in private practice.

The adapted questionnaire consisted of six sections. Section A dealt with the background information and demographics of the participants. Section B contained the knowledge items. This included 61-point knowledge-testing questions. Four of these questions were not applicable to the enrolled nurses, enrolled nursing assistants and CHWs; hence, these cadres responded to 57 knowledge-testing questions. The questions were mainly a combination of the following formats: single best answer; multiple choice; ‘list’ type; ‘yes’, ‘no’ or ‘I don’t know’; ‘true’, ‘false’ or ‘I don’t know’. The correct and incorrect responses were scored 1 and 0, respectively. The aspects of PCa knowledge tested included: function of the prostate, risk factors, symptoms, screening tests, screening test interpretation, diagnosis, staging, treatment and complications of treatment.

Bloom’s cut-off points^36^ was used to categorise knowledge levels as follows:

- good knowledge (80% – 100% correct responses)
- moderate knowledge (60% – 79% correct responses)
- poor knowledge (< 60% correct responses).

Section C was the attitude section. Participants were requested to respond to 14 statements, through which their attitude towards PCa was measured on a 7-point Likert scale (1 = *strongly disagree*; 7 = *strongly agree*). The individual points per statement were summed up, and the possible obtainable scores ranged from 0 to 98.

Using Bloom’s cut-off points,^36^ the scores were classified as follows:

- positive attitude (80% – 100% correct responses)
- neutral attitude (60% – 79% correct responses)
- negative attitude (< 60% correct responses).

In Section D, the participants’ practices regarding PCa counselling and screening were measured on a 7-point Likert scale. An additional yes-or-no question asked the male participants if they would be willing to have a PCa screening.

The participants responded to nine practice statements. The enrolled nurses, enrolled nursing assistants and CHWs were exempted from three of these statements, which do not apply to them. The practice statements were graded on a 7-point Likert scale (1 = *very untrue of me*; 7 = *very true of me*). The individual points per statement were summed up, and the possible obtainable scores ranged from 0–63 (0–42 for enrolled nurses, enrolled nursing assistants and CHWs)

Using Bloom’s cut-off points,^36^ the scores were classified as follows:

- good practice (80% – 100% of the possible obtainable scores)
- fair practice (60% – 79% of the possible obtainable scores)
- poor practice (< 60% of the possible obtainable scores).

Section E assessed participants’ perceived barriers to PCa counselling and screening. This was measured on a 3-point scale (*true, false, I don’t know*).

Finally, Section F checked the participants’ perceived need for additional knowledge on PCa as well as their preferred method of knowledge transfer.

### Content validity and reliability of the questionnaire

This original questionnaire was validated by experts specialising in urology, public health, health education and behavioural sciences in Saudi Arabia. Cronbach’s reliability test was used to test the internal consistency of the different scales used (the scores were 0.75 for knowledge, 0.65 for attitude and 0.93 for self-efficacy and practice).^34,35^

Changes were made to the original questionnaire to adapt the questions to the study setting, for example, ‘Saudi Arabian men’ was changed to ‘South African men’. The adapted questionnaire was reviewed and approved by a Health Sciences Faculty evaluation committee comprising consultant family physicians, a urologist, medical educators, a professional nurse and a biostatistician.

### Pilot study

The adapted questionnaire was pretested in December 2020 on 22 participants, including two clinical associates and four from each of the other cadres of PHC workers. Participants were chosen in succession. The pilot study was to ensure that the questions were balanced and correctly constructed and that the crucial information was obtained. The 22 piloted questionnaires were included in the study because no significant changes arose from the pilot study.

### Data analysis

The data were analysed by the Department of Biostatistics, Faculty of Health Sciences, University of the Free State (UFS), using Statistical Analysis System (SAS) version 9 (SAS Institute Inc., Cary, North Carolina, U.S.). Descriptive statistics (e.g. median and standard deviation [s.d.]) were used for continuous variables, while frequencies and percentages were calculated for categorical data. Association between variables were assessed using chi-square or Fisher’s exact tests. A level of significance was set at *p*-value of < 0.05.

### Ethical considerations

The permission to conduct the study was granted by the head of the Free State Department of Health. Ethical approval for the study was obtained from the Health Sciences Research Ethics Committee of the University of the Free State (ethical clearance number UFS-HSD2020/1481/2411).

Before study participation, each participant gave informed consent after being provided with a detailed description of the study. The voluntary nature of participation and the right to refuse participation or withdraw during the study was explained. The self-administered questionnaire was anonymous. The names of participants were not recorded on any of the documents.

## Results

### Sociodemographic characteristics of participants

Five hundred and forty-eight (*n* = 548) PHC providers participated out of the 763 eligible participants invited, giving a response rate of 71.8%. Table 1 summarises the participant characteristics.

**TABLE 1:** Sociodemographic characteristics of participants (*n* = 548).

Variable	*n*	%
**Gender**		
Female	418	76.3
Male	130	23.7
**Age group (years)**		
22–30	157	28.6
31–40	159	29.0
41–50	121	22.1
51–60	100	18.2
> 60	11	2.0
**Profession or rank**		
Family physician	17	3.1
Medical officer or GP	88	16.1
Community service medical officer	36	6.6
Medical intern	26	4.7
Professional nurse	142	25.9
Enrolled nurse	27	4.9
Enrolled nursing assistant	55	10.0
Clinical associate	8	1.5
Community health worker	149	27.2
**Work sector**		
State	505	92.2
Private	26	4.7
Both	17	3.1
**Years of experience in current position**		
< 1 year	47	8.6
1–5 years	216	39.4
6–10 years	106	19.3
> 10 years	179	32.7
**Additional postgraduate qualification**		
Yes	110	20.1
No	438	79.9

GP, general practitioner.

The median age of the participants was 38 years (range 22–77 years). Of the 309 participants with a degree(s) (or equivalent), 110 (35.6%) had postgraduate qualifications, ranging from postgraduate certificates to PhD degrees. Forty-three (7.8%) participants had some training outside South Africa, while 39 (7.1%) had practised outside South Africa. Forty-one (7.5%) participants had held posts or worked in a urology unit, the duration of which was less than a year (*n* = 33, 80.5%). Most (*n* = 511, 93.2%) participants had never attended continuing medical education (CME) focusing on PCa. Only 28 (5.1%) worked in a facility that runs a men’s health clinic. Sixty-nine (12.6%) participants were involved in the training of medical students.

### Other prostate cancer–related background information

About a fifth (*n* = 117, 21.4%) of the participants had guidelines on PCa screening, and 192 (35.0%) were aware of the South African Prostate Diagnostic and Treatment Guidelines, 2017 (SAPDTG).^11^ A total of 207 (37.8%) participants had either given a health talk about PCa or conducted PCa screening with PSA in the past. These participants’ (self-reported) practice of SDM is shown in Table 2.

**TABLE 2:** Participants’ (self-reported) practice of shared-decision making (*n* = 207).

Shared-decision making criteria	Participants’ (self-reported) practice					
	Never		Partially		Fully	
	*n*	%	*n*	%	*n*	%
I discussed the advantages of the screening blood test with my patients	26	12.6	99	47.8	82	39.6
I discussed the disadvantages of the screening blood test with my patients	94	45.4	69	33.3	44	21.3
I informed my patients that some experts disagree about whether men should have prostate-specific antigen test or not	143	69.1	50	24.2	14	6.8

### Assessment of participants’ knowledge about prostate cancer

The majority (*n* = 355, 64.8%) of participants had poor knowledge (< 60%), 30.1% (*n* = 165) had moderate knowledge (60% – 79%), while 5.1% (*n* = 28) had good knowledge (80% – 100%). However, 274 (50.0%) participants had a score of ≥ 50%. The mean knowledge scores (± s.d.) per profession are shown in Table 3.

**TABLE 3:** Knowledge scores per profession or rank (*n* = 548).

Profession	Mean knowledge score (%)	± s.d.	Minimum (%)	Maximum (%)
Family physician	70.1	± 8.95	46	85
Medical officer or GP	67.1	± 12.47	28	93
Community service medical officer	66.4	± 11.6	38	85
Intern	70.7	± 10.02	46	87
Professional nurse	51.1	± 15.45	2	80
Enrolled nurse	36.4	± 25.93	0	77
Enrolled nursing assistant	33.1	± 20.26	0	84
Clinical associate	53.4	± 21.78	13	80
Community health worker	26.1	± 20.37	0	80

GP, general practitioner; s.d., standard deviation.

#### Comparison between different background characteristics and participants’ knowledge

As shown in Table 6, compared with the male participants, more women had poor knowledge. Medical officers or GPs had better knowledge compared with other professions and ranks (*p* < 0.001). More state-employed participants had better knowledge than those in the private sector or both sectors (*p* < 0.001). Participants with over 10 years’ working experience had better knowledge, although it was not statistically significant (*p* = 0.064).

Participants without prior working experience in urology had poor knowledge (*p* = 0.001). Participants who had never attended PCa-related CME or continuing professional development (CPD) had poor knowledge (*p* < 0.001). Running men’s health clinics did not show any statistically significant association with the participants’ knowledge (*p* = 0.062). Participants uninvolved with the training of medical students had poor knowledge compared with those involved in providing such training (*p* < 0.001).

Participants unaware of the SAPDTG^11^ had poor knowledge (*p* < 0.001). Participants without PCa screening guidelines in their practice had poor knowledge (*p* = 0.044). Participants who had previously conducted PCa screening or given relevant health talks had better knowledge than those who had not (*p* < 0.001).

### Assessment of participants’ attitudes towards prostate cancer screening

Most (*n* = 321, 58.6%) participants had a neutral attitude (60% – 79%), 40.7% (*n* = 223) had a negative attitude (< 60%), while 0.7% (*n* = 4) had a positive attitude (80% – 100%). Table 4 shows the level of agreement to the statements regarding attitude towards PCa.

**TABLE 4:** Participants’ attitudes regarding prostate cancer (*n* = 548).

Attitude statement	Strongly disagree (%)	Disagree (%)	Somewhat disagree (%)	Neutral (%)	Somewhat agree (%)	Agree (%)	Strongly agree (%)
1. Early detection through screening can improve survival for men with PCa	5.8	2.6	0.9	6.4	5.8	20.6	57.8
2. PCa counselling and screening should be routinely used on all men beginning at age 50	5.3	5.1	1.5	8.6	8.0	26.1	45.4
3. The DRE is an accurate screening test for PCa	3.6	6.0	5.3	20.4	16.4	25.9	22.3
4. There is evidence to support using DRE for PCa screening on asymptomatic men with no risk factors	6.0	9.7	3.8	29.6	14.1	23.0	13.9
5. The DRE is unaccepted by South African men, so PHC practitioners should avoid it	29.4	22.1	5.1	23.7	7.8	7.3	4.6
6. I am uncomfortable with practice relating to men’s health	24.3	21.7	6.2	23.5	8.0	10.9	5.3
7. The PSA is an accurate screening test for PCa	3.5	7.8	5.1	27.4	14.2	21.9	20.1
8. There is enough evidence to support using PSA for PCa screening on asymptomatic men with no risk factors	5.1	11.3	7.5	28.5	13.7	19.3	14.6
9. It is more appropriate for specialists to screen for PCa	13.0	19.7	5.7	22.1	7.7	15.9	16.1
10. I think that PSA testing leads to excess subsequent unnecessary investigations	14.6	23.5	8.6	28.1	11.7	7.5	6.0
11. The DRE and serum PSA screening of asymptomatic men reduces PCa mortality	4.9	7.8	4.4	27.2	11.1	22.4	22.1
12. Patients with history of lower urinary tract symptoms and clinical suspicion of PCa should have their PSA tested	2.0	3.8	3.1	19.9	12.0	28.5	30.7
13. I will conduct PCa screening on any man requesting it	5.5	8.8	5.5	20.3	10.0	21.5	28.5
14. The PCa screening is unnecessary in men > 70 years	37.2	22.4	5.3	18.2	6.4	6.0	4.4

DRE, digital rectal examination; PCa, prostate cancer; PHC, primary health care; PSA, prostate-specific antigen.

#### Comparison between different background characteristics and participants’ attitudes

The female participants were more uncomfortable with practice relating to men’s health than their male counterparts (*p* = 0.003). Professional nurses and CHWs were more uncomfortable with practice pertaining to men’s health compared with participants of other professions and ranks (*p* = 0.002). More CHWs had a negative attitude than participants of other professions or ranks (*p* < 0.001).

The bivariate analysis in Table 6 shows that more female participants had a negative attitude compared with their male counterparts. More state-employed participants had a positive attitude compared with those of other work sectors (*p* < 0.001). Participants with 1–5 years’ working experience had a positive attitude compared with those with other years of experience (*p* = 0.014). Participants without additional postgraduate qualifications (compared with those who have) had a negative attitude, although this was not of statistical significance (*p* = 0.105).

Participants without previous work experience in urology had a negative attitude compared with those with prior urology work experience, although this association was not of statistical significance (*p* = 0.239). Participants who had never attended PCa-related CME or CPD had a negative attitude (*p* = 0.047).

More participants aware of the SAPDTG^11^ had a positive attitude than those unaware of the guidelines (*p* = 0.027). More participants with poor knowledge had a negative attitude than participants in the other knowledge categories (*p* < 0.001).

### Assessment of participants’ practice regarding prostate cancer screening and counselling

The majority (*n* = 219, 40.0%) of participants had poor practice (< 60%), 35.8% (*n* = 196) had fair practice (60% – 79%), while 24.3% (*n* = 133) had good practice (80% – 100%). Table 5 shows the participants’ self-reported level of confidence regarding PCa screening and counselling practices.

**TABLE 5:** Participants’ practices regarding prostate cancer screening and counselling (*n* = 548).

Please rate your confidence on your ability to perform the following:	Very untrue of me (%)	Untrue of me (%)	Somewhat untrue of me (%)	Neutral (%)	Somewhat true of me (%)	True of me (%)	Very true of me (%)
1. I am able to provide effective counselling of asymptomatic men on PCa	9.3	10.2	5.3	19.3	22.1	21.4	12.4
2. I am able to take a proper history in order to identify risk factors and symptoms of PCa from patients	7.3	9.3	3.5	20.3	21.5	22.8	15.3
3. I am able to refer patients at high risk for PCa for screening	7.3	7.5	5.8	17.9	12.6	24.8	24.1
4. I am able to follow up patients at high risk for PCa	9.7	10.6	7.3	23.4	12.4	20.8	15.9
5. I am able to examine the prostate by DRE†	21.5	6.9	4.7	11.4	6.3	28.1	21.1
6. I am able to detect palpable abnormalities on the prostate during DRE†	19.2	9.5	3.2	9.8	12.3	29.3	16.7
7. I am able to find suitable options for treatment of patients with PCa†	17.4	12.3	6.6	20.2	11.7	22.7	9.1
8. I am able to counsel patients on the benefits of PSA testing	14.6	10.8	5.3	22.8	15.5	20.6	10.4
9. I am able to discuss the various treatment modalities of PCa with my patients	19.3	12.8	6.2	25.2	13.7	13.9	8.9

DRE, digital rectal examination; PCa, prostate cancer; PSA, prostate-specific antigen.

†, enrolled nurses, enrolled nursing assistants and community health workers (CHW) were exempted from practice items 5–7 as these are beyond their scope of practice.

Male participants tended to be more comfortable with DRE than their female counterparts (*p* < 0.001). Unlike the other professions or ranks, the professional nurses were less comfortable with DRE (*p* < 0.001) and felt less competent in detecting abnormalities on DRE (*p* < 0.001). Of the 130 male participants, 100 (75.8%) would consider having PCa screening on themselves.

#### Comparison between different background characteristics and participants’ practices

As shown in Table 6, compared with their male counterparts, more female participants had poor practice (*p* < 0.001). Medical officers or GPs had good practice compared with the other professions or ranks (*p* < 0.001). State-employed participants had good practice in comparison to those of other work sectors (*p* < 0.001). Participants with 1–5 years’ working experience had good practice compared with those with other years of experience (*p* = 0.019). Participants without additional postgraduate qualifications (compared with those who have) had poor practice (*p* = 0.041).

**TABLE 6:** Bivariate analysis of background characteristics versus knowledge, attitude and practice.

Background characteristic	Good knowledge (*n* = 28)		Moderate knowledge (*n* = 165)		Poor knowledge (*n* = 355)		*P-*value	Positive attitude (*n* = 4)		Neutral attitude (*n* = 321)		Negative attitude (*n* = 223)		*P*-value	Good practice (*n* = 133)		Fair practice (*n* = 196)		Poor practice (*n* = 219)		*P*-value
	*n*	%	*n*	%	*n*	%		*n*	%	*n*	%	*n*	%		*n*	%	*n*	%	*n*	%	
**Gender**							< 0.001†							0.029†							< 0.001†
Male	14	50.0	66	40.0	50	14.1		2	50.0	87	27.1	41	18.4		55	41.4	44	22.4	31	14.2	
Female	14	50.0	99	60.0	305	85.9		2	50.0	234	72.9	182	81.6		78	58.6	152	77.6	188	85.8	
**Work sector**							< 0.001†							< 0.001†							< 0.001†
State	23	82.1	141	85.5	340	96.0		2	50.0	284	88.5	218	98.2		111	84.1	181	92.3	212	96.8	
Private	4	14.3	15	9.1	7	2.0		1	25.0	24	7.5	1	0.5		15	11.4	9	4.6	2	0.9	
State and private	1	3.6	9	5.5	7	2.0		1	25.0	13	4.0	3	1.4		6	4.5	6	3.1	5	2.3	
**YOWE**							0.064							0.014†							0.019†
< 1 year	0	0	7	4.2	40	11.3		0	0	20	6.2	27	12.1		7	5.3	14	7.1	26	11.9	
1–5 years	11	39.3	71	43.0	134	37.7		2	50.0	134	41.7	80	35.9		51	38.3	94	48.0	71	32.4	
6–10 years	5	17.9	29	17.6	72	20.3		1	25.0	50	15.6	55	24.7		31	23.3	32	16.3	43	19.6	
> 10 years	12	42.9	58	35.2	109	30.7		1	25.0	117	36.4	61	27.4		44	33.1	56	28.6	79	36.1	
**Additional qualifications**							0.003†							0.105							0.041†
Yes	9	32.1	45	27.3	56	15.8		1	25.0	74	23.1	35	15.7		31	23.3	28	14.3	51	23.3	
No	19	67.9	120	72.7	299	84.2		3	75.0	247	76.9	188	84.3		102	76.7	168	85.7	168	76.7	
**CME or CPD**							< 0.001†							0.047†							< 0.001†
Yes	6	21.4	23	13.9	8	2.3		1	25.0	27	8.4	9	4.0		18	13.5	14	7.1	5	2.3	
No	22	78.6	142	86.1	347	97.7		3	75.0	294	91.6	214	96.0		115	86.5	182	92.9	214	97.7	
**Men’s clinic**							0.062							0.193							0.005
Yes	1	3.6	14	8.5	13	3.7		1	25.0	16	5.0	11	4.9		11	8.3	14	7.1	3	1.4	
No	27	96.4	151	91.5	342	96.3		3	75.0	305	95.0	212	95.1		122	91.7	182	92.9	216	98.6	
**Training UG or PG students**							< 0.001†							0.285							< 0.001†
Yes	9	32.1	38	23.0	22	6.2		0	0	46	14.3	23	10.3		33	24.8	23	11.7	13	5.9	
No	19	67.9	127	77.0	333	93.8		4	100.0	275	85.7	200	89.7		100	75.2	173	88.3	206	94.1	
**Aware of SADTG**							< 0.001†							0.027†							< 0.001†
Yes	12	42.9	83	50.3	97	27.3		3	75.0	123	38.3	66	29.6		69	51.9	74	37.8	49	22.4	
No	16	57.1	82	49.7	258	72.7		1	25.0	198	61.7	157	70.4		64	48.1	122	62.2	170	77.6	
**Screening guidelines**							0.044†							0.271							0.001†
Yes	10	35.7	41	24.8	66	18.6		1	25.0	76	23.7	40	17.9		40	30.1	46	23.5	31	14.2	
No	18	64.3	124	75.2	289	81.4		3	75.0	245	76.3	183	82.1		93	69.9	150	76.5	188	85.8	
**Did previous screening**							< 0.001†							0.382							< 0.001†
Yes	21	75.0	83	50.3	103	29.1		2	50.0	128	40.0	77	34.5		68	51.5	84	42.9	55	25.1	
No	7	25.0	82	49.7	251	70.9		2	50.0	192	60.0	146	65.5		64	48.5	112	57.1	164	74.9	

CME, continuing medical education; CPD, continuing professional development; PG, postgraduate; SAPDTG, South African Prostate Diagnostic and Treatment Guidelines^11^ UG, undergraduates; YOWE, years of working experience. z, Values are indicative of a statistically significant association.

Participants who had never attended PCa-related CME or CPD had poor practice (*p* < 0.001). Participants without a men’s clinic in their practice had poorer practice than those having such a clinic (*p* = 0.005). Participants uninvolved with training of medical students had poorer practice than those involved in giving such training (*p* < 0.001).

Participants aware of the SAPDTG^11^ had better practice than those unaware of the guidelines (*p* < 0.001). Participants without PCa screening guidelines in their practice had poorer practice than those with such guidelines (*p* < 0.001). Participants who had previously conducted PCa screening or given relevant health talks had good practice compared with those who had not (*p* < 0.001).

### Overall comparison between knowledge, attitude and practice

Participants with poor knowledge had poor practice compared with other knowledge categories (*p* < 0.001). Participants who had a negative attitude had poor practice compared with other attitude categories (*p* < 0.001).

### Perceived barriers to prostate cancer screening and counselling

Table 7 shows the participants’ perceived barriers to PCa counselling and screening. Most participants reported a lack of knowledge among healthcare practitioners and men at risk as possible barriers.

**TABLE 7:** Perceived barriers to prostate cancer screening and counselling (*n* = 548).

Possible barriers to prostate cancer counselling and screening	True (%)	False (%)	I don’t know (%)
A lack of knowledge among healthcare practitioners	74.8	3.6	21.5
A lack of knowledge among ‘at risk’ men	75.5	5.3	19.2
Inadequate skills among healthcare practitioners	65.4	8.8	25.8
Refusal of patients	57.5	15.1	27.4
Screening tests are inaccurate	28.7	38.8	32.5
Prostate cancer is not a public problem	22.1	57.0	20.8

### Need for more knowledge relating to prostate cancer

The majority (*n* = 489, 89.4%) of participants felt they needed additional knowledge regarding PCa screening. These aspects included: treatment (*n* = 392, 80.2%), risk factors and counselling (*n* = 387, 79.1%), value of nutrients (*n* = 367, 75.1%), diagnosis (*n* = 351, 71.8%), symptoms (*n* = 345, 70.6%), value of PSA testing (*n* = 338, 69.1%) and value of DRE (*n* = 335, 68.5%).

The preferred methods of knowledge delivery were as follows: study material (*n* = 374, 76.5%), practical sessions (*n* = 312, 63.8%), simulated scenarios (*n* = 288, 58.9%), group tasks (*n* = 254, 51.9%), didactic lectures (*n* = 218, 44.6%) and other – online CPD (*n* = 8, 1.6%).

## Discussion

### Sociodemographic and background characteristics

The majority (76.3%) of the participants were women. This may be because PHC clinics in South Africa are nurse-driven. Most nurses and CHWs were women; these two cadres of PHC providers formed about 60% of the participants in this study. The majority of the participants were in the age group 31–40 years old, with a median age of 38 years. The majority (39.4%) had work experience of 1–5 years. Among the medical doctors in this study, the majority were medical officers. Similar demographic patterns were found in a study^37^ conducted among medical doctors and nurses in the Free State, where the majority were women and nurses, in the age group 30–39 with a mean age of 39 years, with between 1–5 years’ experience, and the majority of the medical doctors were medical officers.

The majority of the participants had never worked in facilities running men’s health clinics (94.9%). Most participants (93.2%) had never attended CME related to PCa. This may suggest that this subject and perhaps subjects relating to men’s health have not been prioritised among some PHC providers in the study setting.^38^ This subject of men’s health is also unlikely to have been prioritised when it comes to refresher courses considered for PHC providers.

About a fifth (21.4%) of the participants had guidelines on PCa screening at their practice, and 35% were aware of the SAPDTG.^11^ This approximately correlates with the 37.8% who had ever either given a health talk on PCa or conducted a PCa screening with PSA. However, as shown in Table 2, it is unlikely that the participants were aware of the proper conduct of SDM. There is therefore a need for PHC providers to be acquainted with the steps engaged in the conduct of SDM and be trained on how to practise them in sync with good consultation.^22^

### Knowledge, attitude and practice of participants on prostate cancer screening and early diagnosis

#### Knowledge

Almost two-thirds (64.8%) of the participants in this study had poor knowledge. In a similar study^35^ among primary care physicians in Saudi Arabia, the mean knowledge score was 54.3%. The overall poor knowledge among the participants in the present study may be because of the varied cadre of PHC providers with varied levels of knowledge. As seen in Table 3, the mean knowledge score for family physicians was 70.1% compared with CHWs with 26.1%. Diverse questions may arise from patients during the process of SDM. Primary health care providers’ sound knowledge of the screening tests (and their interpretations), diagnosis, investigations, treatment modalities, complications (of the disease and treatment), among others, is essential to conduct an efficient SDM process.

Good knowledge had a statistically significant association with the following factors: state-employed PHC providers (*p* < 0.001), being a GP or medical officer (*p* < 0.001) and having previously conducted PCa screening or given relevant health talks (*p* < 0.001). Reiterative education and practice of this subject is therefore important.

Poor knowledge, on the other hand, had a statistically significant association with the following factors: female PHC providers (*p* < 0.001), no previous CME on PCa topics (*p* < 0.001), uninvolved in medical student training (*p* < 0.001), unaware of the SAPDTG^11^ (*p* < 0.001) and no PCa screening guidelines at practice site (*p* = 0.044). The same explanation (as given) holds; a lack of opportunities and activities promoting reiterative education on this subject may gradually impact negatively on the knowledge.

#### Attitude

There was an overall prevalence of a neutral attitude (58.6%) among the participants. In a Saudi Arabian study,^35^ the mean total attitude score was greater than the midpoint. In the same Saudi Arabian study,^35^ most participants believed that early detection of PCa through screening could improve the survival of men. Similarly, the majority of the participants in this study believed there is value in early diagnosis through screening. However, about 40% of the participants felt specialists should perform PCa screening, as observed from their response to the following statement: ‘It is more appropriate for specialists to screen for PCa’. This is unlike the finding in the Saudi Arabian study,^35^ where most of the participants accepted PCa screening as their role.

Participants’ responses to the statements, ‘I will conduct PCa screening on any man requesting it’ and ‘PCa screening is unnecessary in men > 70 years’ could imply that some of them may engage in an irrational screening approach. Also, there seems to be an inadequate understanding of the use and interpretation of the screening tests, as the majority of the participants felt that PSA and DRE are accurate screening tests for PCa.

Most of the participants agreed to the following statements: ‘There is evidence to support using DRE for PCa screening on asymptomatic men with no risk factors’ and ‘There is enough evidence to support using PSA for PCa screening on asymptomatic men with no risk factors’. This is in contrast to a study^34^ carried out among physicians, where only about a fifth agreed to these statements. Primary health care provider information provided to patients during SDM could therefore be questionable, in the study setting.

Over 40% of the participants were either neutral or agreed to the statement, ‘DRE is unaccepted by South African men, so PHC practitioners should avoid it’. This belief might have informed their response to the statement, ‘I am uncomfortable with practice relating to men’s health’, to which over 40% either agreed or were neutral. Being a female PHC provider was associated with being uncomfortable with practices relating to men’s health (*p* = 0.003).

Factors found to be significantly associated with a positive attitude towards PCa screening include state-employed PHC providers (*p* < 0.001), 1–5 years’ working experience (*p* = 0.014) and awareness of the SAPDTG^11^ (*p* = 0.027). Factors associated with a negative attitude towards PCa screening include being a female PHC provider (*p* = 0.029), no previous CME on PCa topic (*p* = 0.047) and poor knowledge of PCa screening (*p* < 0.001). As stated earlier, female PHC providers (mostly nurses) are gradually avoiding certain clinical functions relating to men’s health. This may be because of certain perceptions and beliefs, particularly PHC provider and patient gender differences. This is further compromised by the scarcity of continued healthcare education on this subject. Knowledge was a top determinant of attitude towards PCa counselling and screening, as confirmed in a similar study by Arafa et al.^35^

#### Practice

There was an overall prevalence of poor practice (40%) among the participants. The responses of the participants to the following practice statements: ‘I am able to counsel patients on the benefits of PSA testing’, and ‘I am able to discuss the various treatment modalities of PCa with my patients’ could imply their inability to conduct an effective SDM process; this is also reflected in their self-reported practice on SDM (Table 2). In a similar study,^26^ 60% of the primary care physicians would discuss the implication of an abnormal PSA test before performing it, and 20% would discuss the treatment modalities for PCa before screening. There may be the need to review the consultation styles of PHC providers in this setting.

Almost 60% of the participants responded positively to the practice statement, ‘I am able to take a proper history in order to identify risk factors and symptoms of PCa from patients’; this is a reflection of their satisfactory knowledge on the risk factors and symptoms of PCa.

The majority of the participants who responded negatively to the practice statement ‘I am able to examine the prostate by DRE’ and ‘I am able to detect palpable abnormalities on the prostate during DRE’ were nurses. According to the former head, School of Nursing, UFS (Prof. Magda Mulder 2020, personal communication, June 13), nurses’ practice of DRE is gradually fading, despite being within their scope of practice. Time constraints running busy clinics might hinder this practice in the study setting. With proper training of PHC providers (especially nurses) and time management, opportunistic screening in the form of annual DRE and PSA checks can be included in men’s regular care.

More than three-quarters (75.8%) of the male participants in this study would consider having PCa screening on themselves. In a similar study,^26^ almost 90% of the participants would consider going for a PSA test. Factors found to be significantly associated with good practice of PCa screening include: being a state-employed PHC provider (*p* < 0.001), 1–5 years working experience (*p* < 0.001), being a medical officer or GP (private practice) (*p* < 0.001), having additional postgraduate qualifications (*p* = 0.041), awareness of the SAPDTG^11^ (*p* < 0.001) and previous conduct of PCa screening (*p* < 0.001).

Factors found to be significantly associated with poor practice include: no previous CME on the topic of PCa (*p* < 0.001), not having a men’s clinic in practice (*p* = 0.005), being uninvolved with medical student training (*p* < 0.001), no PCa screening guidelines in the practice (*p* < 0.001), poor knowledge on PCa screening (*p* < 0.001) and a negative attitude towards PCa screening (*p* < 0.001). In a similar study,^26^ factors associated with an inclination of the primary care physician to screen men for PCa were the older age group, longer duration of practice and those who would consider having PSA conducted on themselves.

### Barriers to prostate cancer counselling and screening

Top self-reported barriers to PCa counselling and screening were a lack of knowledge among healthcare practitioners and ‘at risk’ men and inadequate skills among healthcare practitioners. In a similar study,^35^ apart from the lack of skills and knowledge, refusal of patients and PCa not being viewed as a public health concern were the barriers to screening and counselling for the disease.

### Need for additional learning

The majority of the participants felt the need for additional learning in the following topics relating to PCa: (1) risk factors and counselling, (2) symptoms, (3) the value of nutrients, (4) the value of PSA testing, (5) the value of DRE, (6) diagnosis and (7) treatment. The preferred methods of instruction suggested by the majority included study materials, practical sessions, simulated scenarios and group tasks.

Continuing professional development refers to the education that follows certification and licensure and has been shown to improve healthcare provider practice and, in some instances, healthcare outcomes.^39^ Experts suggest the need for the development of more effective courses for PHC providers (especially the nurses, clinical associates and CHWs) to enhance their educational background, thereby improving their collaborative functionality within the PHC system.^40,41^

While the importance of continued healthcare provider education has been described, there is a lack of research on the specific strategies to provide this education. The following have been shown as effective methods of instruction among CHWs: (1) role play, (2) case studies, (3) teach-back, (4) group presentation, (5) interactive didactic, (6) demonstration, (7) group assessment, (8) group discussion, (9) hands-on practice and (10) individual assessment.^42^ In the nursing profession, commonly used practices for continuing nursing education are short, repeated education; the use of interactive techniques, for example, simulation models; audio-visual learning modalities; teamwork in healthcare settings; use of cultural context; and the practice of assessment and feedback.^43^

To maximise the outcome of CPD activities, the preferred learning styles and methods of the targeted audience need to be considered. A survey among PHC physicians showed a higher preference for learning about cancer screening through the following educational formats: conferences; self-directed, small group workshops; hospital rounds; and online CME and CPD.^39^

### Recommendations

The identified KAP gaps and the self-reported additional knowledge needs should be addressed in the medical undergraduate curriculum and CPD activities and refresher courses developed for practising PHC providers, bearing in mind their preferred learning methods. Women constitute the majority among the PHC providers in the study setting; measures should be put in place to improve their confidence during PCa counselling and screening and other practices relating to men’s health.

Community health workers in conjunction with the Ward-Based Outreach Team (WBOT) provide PHC services (including health education and promotion) from household to household; there is therefore the need to channel adequate resources towards their proper training, mentoring and coordination. Family physicians who are champions of PHC should be saddled with the coordination and provision of these teaching and learning needs in the community.

### Strengths and limitations

To the best of our knowledge, this is the first KAP study on PCa among a wide range of PHC providers in South Africa. This study offered the participants the opportunity to determine their learning needs as well as their preferred learning methods on the subject of PCa; the study’s findings may therefore be a valuable tool in planning the needful intervention.

Study limitations include that the study was conducted among PHC providers in the Free State; therefore, it may not be generalised to other populations or other specialised healthcare providers in the country. The ongoing coronavirus disease 2019 (COVID-19) pandemic limited access to GPs in private practice. As a result of the diversified cadres of healthcare providers, multiple sampling methods were engaged in this study; some degree of selection bias is possible. Of note is the unequal gender distribution among participants in this study, with women dominating; this likely affected some of the bivariate analysis. Although the questionnaire items were basic and unambiguous, the possibility of information bias should be borne in mind because of the diverse scope of practice among various cadres of healthcare providers. Lastly, the use of self-reported data in some aspects of the study made it prone to recall bias.

## Conclusions

Women, who constituted the majority of the participating PHC providers in this study, have appreciable KAP gaps relating to PCa screening. There is also a likelihood of poor knowledge on the proper conduct of SDM, which is an important subject and prerequisite, owing to the controversies regarding PCa screening; therefore, there is the likelihood of improper conduct of PCa screening. The participants’ perceived need for additional knowledge on the subject and preferred learning methods were determined. Factors associated with their KAP were also identified, which indicated the need for reiterative learning and teaching on this subject.
